# Diastolic function evaluation in children with ventricular arrhythmia

**DOI:** 10.1038/s41598-023-33118-x

**Published:** 2023-04-11

**Authors:** Radosław Pietrzak, Tomasz M. Książczyk, Magda Franke, Bożena Werner

**Affiliations:** Department of Pediatric Cardiology and General Pediatrics, Medical University of Warsaw, Żwirki I Wigury Street 61, 02-091 Warszawa, Poland

**Keywords:** Cardiology, Health care, Medical research, Signs and symptoms

## Abstract

Premature ventricular contractions (PVC) are frequently seen in children. We evaluated left ventricular diastolic function in PVC children with normal left ventricular systolic function to detect whether diastolic function disturbances affect physical performance. The study group consisted of 36 PVC children, and the control group comprised 33 healthy volunteers. Echocardiographic diastolic function parameters such as left atrial volume index (LAVI), left atrial strains (AC-R, AC-CT, AC-CD), E wave, E deceleration time (Edt), E/E’ ratio, and isovolumic relaxation time (IVRT) were measured. In the cardiopulmonary exercise test (CPET), oxygen uptake (VO_2 max_) was registered. Evaluation of diastolic function parameters revealed statically significant differences between the patients and controls regarding Edt (176.58 ± 54.8 ms vs. 136.94 ± 27.8 ms, *p* < 0.01), E/E’ (12.6 ± 3.0 vs. 6.7 ± 1.0, *p* < 0.01), and IVRT (96.6 ± 19.09 ms. vs. 72.86 ± 13.67 ms, *p* < 0.01). Left atrial function was impaired in the study group compared to controls: LAVI (25.3 ± 8.2 ml/m^2^ vs. 19.2 ± 7.5 ml/m^2^, *p* < 0.01), AC-CT (34.8 ± 8.6% vs. 44.8 ± 11.8%, *p* < 0.01), and AC-R-(6.0 ± 4.9% vs. −11.5 ± 3.5%, *p* < 0.01), respectively. VO2 max in the study group reached 33.1 ± 6.2 ml/min/kg. A statistically significant, moderate, negative correlation between VO2 max and E/E’ (r = −0.33, *p* = 0.02) was found. Left ventricular diastolic function is impaired and deteriorates with the arrhythmia burden increase in PVC children. Ventricular arrhythmia in young individuals may be related to the filling pressure elevation and drive to exercise capacity deterioration.

## Introduction

Premature ventricular contractions (PVCs) are among the most common rhythm disorders. They are frequently observed in children and adolescents without structural heart disease and are considered benign. However, this hypothesis is uncertain due to existing observations indicating some connections between PVCs and left ventricular systolic dysfunction^1^. Risk factors, such as frequent PVCs, short coupling intervals between the sinus and premature beat, retrograde P-waves, broad QRS complexes, and complex arrhythmia, may induce systolic dysfunction^2–6^.

Moreover, there are data in the literature showing that in adults with PVC and normal ejection fraction, some diastolic function parameters, such as left atrial volume or diastolic filling pressure of the left ventricle, are impaired^7,8^. This issue has not been investigated in children yet. In the recent data from our center, it was detected that exercise capacity is lowered in young individuals with PVC even if the left ventricular systolic function is normal^9^. This finding raises the question: what is the reason for these circumstances? One of the hypotheses is that the reason for low exercise capacity is dyssynchronous contraction. Nevertheless, we know that left ventricular diastolic dysfunction relates to the decrease of exertion tolerance in some clinical scenarios^10–13^.

Considering these data, we aimed to evaluate the left ventricular diastolic function in young individuals with frequent ventricular arrhythmia and normal left ventricular systolic function, to detect whether potential diastolic function disturbances influence physical performance.

## Methods

### Ethical statement

The study for prospective research was approved by the Bioethical Committee of the Medical University of Warsaw, No. 98/2020 dated June 15, 2020. The research reported in this paper adhered to all relevant guidelines and regulations including the Declaration of Helsinki. All the participants and their legal guardians signed an informed consent form before involvement in the study.

The present study was conducted prospectively between January 2022 and June 2022. The study group consisted of 36 patients of both sexes (19 girls, 17 boys), aged 12 to 17 years, in whom extra beats were detected by auscultation or ventricular arrhythmia was registered in ECG evaluation during well-child visits. The primary inclusion criteria were as follows: age range 8–18 years, newly diagnosed, meaning not more than 3 months before the study, frequent ventricular arrhythmia, defined as at least 10% premature ventricular contractions on Holter ECG monitoring, and feasibility to perform cardiopulmonary exercise testing (CPET). Children with pharmacologically treated arrhythmia were excluded from the study group. Thirty-three age- and sex-matched healthy volunteers (18 girls, 15 boys) were enrolled during the same study period as a control group. The overall hours of activity per week were registered during the history taking. Exclusion criteria for both groups were age below 8 years or 18 years and above, any coexisting cardiac disorders or chronic comorbidities not allowing proper CPET performance, and more than 4 h organized physical activity per week. In the physical assessment, the following anthropometrical measurements and vital signs were performed: weight, height, body surface area, body mass index (expressed as z-score), heart rate, and blood pressure.

All patients and controls underwent resting ECG, 24-h Holter monitoring, echocardiography, and CPET.

### ECG assessment

Twelve-lead electrocardiograms were recorded using BioCare ECG (Reynolds Medical System, China). The heart rate, PR, QRS, and QT intervals were measured. QTc was calculated according to the Bazzett formula. The morphology of ventricular extra beats, including the QRS duration, bundle branch block pattern, and the axis of the QRS in limb leads, were assessed. Moreover, 24-h Holter recordings were made using Pathfinder SL (Reynolds Medical System, UK), in which minimal, maximal, and mean heart rates were registered in the study and control group. Additionally, arrhythmia number and burden were registered in the patients. Frequent arrhythmia was defined as a 10% burden in the 24-h Holter ECG. One cardiologist analyzed the recordings, unaware of the subjects’ other data.

### Echocardiography

Conventional transthoracic echocardiography was performed using an EPIQ CVx 5.0 ultrasound machine (Philips Medical System, USA). Two-dimensional guided M-mode, pulsed wave Doppler, tissue Doppler (TDI), and speckle tracking echocardiograms were performed in parasternal short-axis and apical views. The frequencies of the sector array used in the study ranged from 2.1 to 4.2 MHz.

#### Left ventricular assessment

LV end-diastolic dimension (LVDd, mm) and end-systolic dimension (LVSd, mm), interventricular septal diastolic diameter (IVSDd, mm), interventricular septal systolic diameter (IVSSd, mm), left ventricular posterior wall diastolic diameter (LVPWDd, mm), and left ventricular posterior wall systolic diameter (LVPWSd, mm) were measured at the level of the top of the papillary muscles in M-mode imaging. These parameters were indexed to the body surface area and expressed as the z-score (−2, + 2)^14^. Left ventricular ejection fraction (LVEF), representing LV systolic function, was determined using the Simpson method from 4-chamber and 2-chamber apical view imaging. Left ventricular shortness fraction (LVFS, %) was calculated in M-mode imaging. The normal systolic function was defined as a LVDd within the normal limit and EF 55% or more.

#### Mitral valve function assessment

Mitral inflow velocities were obtained from pulsed-wave Doppler in the apical 4-chamber view. Early diastolic mitral inflow velocity (E, cm/s), atrial diastolic mitral inflow velocity (A, cm/s), and E deceleration time (Edt, ms) of the E wave were measured as diastolic echocardiographic variables. Isovolumic relaxation time (IVRT, ms), early diastolic (E’, cm/s), and late diastolic (A’, cm/s) velocities were measured at the septal mitral annulus using the tissue doppler imaging method (TDI). The ratio between E and E’ (E/E’) was calculated as an indicator of LV filling pressure.

#### Left atrial function assessment

Left atrial volume was evaluated using the biplane area-length method from 2 orthogonal apical views, as recommended by the American Society of Echocardiography^15^. It was indexed to body surface area (LAVI—left atrial volume index, ml/m^2^). LA function measures were performed using speckle tracking echocardiography in apical 4-chamber view with the R–R gating. Three variables were assessed. Firstly, the reservoir strain (AC-R, %), the first peak between the R wave and the T wave during left ventricular systole, reflects the left atrial filling. Secondly, the conduit strain (AC-CD, %) reflects the left atrium emptying during the early diastole of the left ventricle. Thirdly, the contractile strain (AC-CT,%), starting on the P wave, reflects the booster pump function of the left atrium. For each parameter, 3 consecutive cardiac cycles were averaged, avoiding PVCs. Analysis of echocardiographic data was performed by an investigator who was unaware of the other data in the study and control group.

### Cardiopulmonary exercise test (CPET)

All patients and controls performed symptom-limited, upright-sitting cardiopulmonary exercise test (CPET) with an incremental RAMP protocol on a bicycle ergometer. After a 3-min rest period, unloaded paddling was made at a rate of 60 rpm for 2 min. The effort was then progressively increased by 10–15 watts/min until the patient could no longer continue a cycling frequency of at least 40 rpm. Cardiopulmonary parameters, such as heart rate, oxygen uptake (VO_2_), and carbon dioxide production (VCO_2_), were continuously monitored. The respiratory exchange ratio (RER) (VCO2/VO2) was simultaneously calculated and used to assess maximal effort. The trial ended when the patient could not maintain the pedal cycle rhythm. No study was interrupted due to cardiopulmonary complications. CPETs were considered valid if the VO_2_ reached a plateau (> 1 min) despite increasing workload and/or a complementary respiratory exchange ratio (RER) was ≥ 1.05 combined with peak heart rate above 85% of predicted. Maximal oxygen uptake (VO2 max) was defined as the oxygen uptake during maximal exercise and was indexed to body weight and time and expressed in milliliters per kilo per minute.

### Statistical analysis

Data were analyzed in R 4.0.5 statistical software (R Core Team [2021]. R: Language and environment for statistical computing by R Foundation for Statistical Computing, Vienna, Austria). Variables were presented as mean ± standard deviation or median (Q1; Q3), depending on data distribution. Distribution normality was assessed using the Shapiro–Wilk test and based on skewness and kurtosis values and visual assessment of histograms. The study and control group were compared with an independent t-test or Mann–Whitney U test, depending on the data distribution. The data were presented as the mean/median difference (MD), including a 95% confidence interval (CI). Additionally, the correlation between continuous variables was verified with Spearman correlation coefficients. To classify the reliability of correlations we used the scale presented in the supplementary file. Table S1^16^. All tests were based on α = 0.05.

## Results

All the patients were newly diagnosed with arrhythmia. The mean age in the study group was 13.8 ± 2 years, and in controls it was 13.2 ± 3 years. None of the children in the study and control group had chronic comorbidities. None of them had clinical signs suggesting arrhythmia presence, and they were not pharmacologically treated when all the tests during the study were performed. Patients and controls had a similar level of everyday organized physical activity of up to 4 h per week at school. Overall physical activity per week was 4.9 ± 1.4 h in the study group and 5.1 ± 1.1 h in the control group, *p* = 0.82. There were no obese children in the study and control group; overweight was seen in one patient of the study group but in none of the control group. The anthropometrical data and vital signs in the study group and controls are presented in Table 1.

**Table 1:** Comparison of anthropometrical parameters and vital signs in the study and control groups.

Characteristic	Study group n = 36 f = 17, m = 19	Control group n = 33 f = 15, m = 18	MD/OR (95% CI)	*p*
Age, years	13.8 ± 2	13.2 ± 3	0.6 (−0.6; 1.8)	0.32
Height (cm)	164 ± 12	163.0 ± 14	1.0 (−5.2; 7.2)	0.92
Weight (kg)	53 ± 15	52 ± 16	3.6 (−6.4; 8.4)	0.88
BSA (m^2^)	1.58 ± 0.30	1.56 ± 0.25	0.0 (−0.12; 0.14)	0.86
BMI (m/kg^2^)	19.9 ± 3.3	20.1 ± 3.2	0.8 (−1.8; 1.4)	0.85
BMI (z-score)	0.1 ± 0.9	0.2 ± 0.4	0.17 (−0.34; 0.34)	0.79
Systlic blood pressure (mmHg)	119 ± 18	117 ± 19	4.5 (−6.9; 10.9)	0.89
Diastolic blood pressure (mmHg)	71 ± 15	68 ± 13	3.4 (−3.8; 9.8)	0.81
HR (bpm)	75 ± 12	77 ± 9	2.7 (−7.5; 3.1)	0.34

*BSA* body surface area, *BMI* body mass index, *OR* odds ratios, *MD* mean/median differences, *f* female, *m* male, *bpm* beats per minute.

### Rest ECG and 24-h holter ECG

In the study group, the median number of PVCs for 24 h was 19,976 beats (mean 14,823 beats), accounting for 20.5 ± 12.1% of the arrhythmia burden. In 28 (78%) patients inferior axis, and in 30 (83%) patients left bundle branch block morphology of PVC was registered. In 8 (22%) and in 6 (17%) patients superior axis and right bundle branch morphology of PVC were seen, respectively. The mean VES QRS duration of PVC was 170 ± 21 ms. In all children of the control group normal sinus rhythm in the ECG and 24-h Holter monitoring was registered, and none of them had ventricular arrhythmia. All the basic parameters during the sinus rhythm in the rest ECG and 24-h Holter ECG are summarized in the supplementary file. Table S2.

### Echocardiography

Left ventricular performance and systolic function.

All the systolic parameters in the study and control group were assessed as normal, and after comparison between the study and control group they were not statistically significant, *p* > 0.5, including LVDd (48.5 ± 4.8 mm vs. 47.4 ± 4.6 mm, respectively), LVDdi (0.5 ± 1.1 vs. 0.0 ± 1.1, respectively), EF (60.0 ± 6.6% vs. 62.2 ± 6.4%, respectively), and SF (39.5 ± 6.0% vs. 40.5 ± 5.5%, respectively). All the data on left ventricular performance and systolic function parameters are summarized in Table 2.

**Table 2:** Echocardiographic parameters of the systolic function in the study and control group.

Echocardiographic parameter	Study group	Control group	MD/OR (95% CI)	*p*
LVDd (mm)	48.5 ± 4.8	47.4 ± 4.6	0.6 (−1.5; 2.7)	0.59
LVDdi (z-score)	0.5 ± 1.1	0.0 ± 1.1	0.3 (−0.03; 1.3)	0.12
LVESd (mm)	29.3 ± 3.4	28.2 ± 3.2	0.8 (−0.4; 2.6)	0.21
LVESdi (z-score)	0.8 ± 1.0	1.0 ± 1.0	23.1 (−47.8; 47.8)	0.3
IVSDd (mm)	7.4 ± 1.4	7.9 ± 1.4	0.3 (−1.1; 0.1)	0.21
IVSDdi (z-score)	0.0 ± 0.7	−0.1 ± 0.3	0.1 (−0.4; 0.2)	0.89
IVSSd (mm)	10.8 ± 1.5	10.0 ± 2.5	0.5 (−0.7; 1.3)	0.58
IVSSdi (z-score)	0.0 ± 0.7	0.1 ± 0.5	0.1 (−0.4; 0.2)	0.61
LVPWDd (mm)	7.4 ± 1.4	7.1 ± 1.1	0.3 (−0.3; 0.9)	0.38
LVPWDdi (z-score)	0.0 ± 0.7	0.1 ± 0.6	0.2 (−0.4; 0.3	0.61
LVPWSd (mm)	11.5 ± 2.1	11.6 ± 2.1	0.5 (−0.9; 1.1)	0.15
LVPWSdi (z-score)	−0.2 ± 0.8	−0.4 ± 0.8	0.2 (−0.2; 0.5)	0.10
LVEF (%)	60.0 ± 6.6	62.2 ± 6.4	−2.2 (−5.3; −0.9)	0.13
LVFS (%)	39.5 ± 6.0	40.5 ± 5.5	1.3 (−3.7; 1.7)	0.17

*LVDd* left ventricular end-diastolic dimension, *LVDdi* left ventricular end-diastolic dimension index, *LVESd* left ventricular end-systolic dimension, *LVESDi *left ventricular end-systolic dimension index, *IVSDd* interventricular septal diastolic diameter, *IVSDd* interventricular septal diastolic diameter index, *IVSSd* interventricular septal systolic diameter, *IVSSd* interventricular septal systolic diameter index, *LVPWDd* left ventricular posterior wall diastolic diameter, *LVPWDd* left ventricular posterior wall diastolic diameter index, *LVPWSd* left ventricular posterior wall systolic diameter, *LVPWSd* left ventricular posterior wall systolic diameter index, *LVEF* left ventricular ejection fraction, *OR* odds ratios, *MD* mean/median differences.

### Diastolic function

A comparison of the study group vs. controls regarding diastolic and left atrial parameters is summarized in Table 3. The analysis revealed a significant difference in E wave deceleration time between PVC children and healthy volunteers (176.58 ± 54.80 ms vs. 136.94 ± 27.83 ms, respectively, *p* < 0.01). Evaluation of tissue Doppler imaging (TDI) parameters showed significant (*p* < 0.01) differences between the study group and controls regarding IVRT (96.60 ± 19.09 ms vs. 72.86 ± 13.67 ms, respectively) and E/E’ ratio (7.60 ± 1.81 vs. 6.73 ± 1.05, respectively). The differences between the study and control groups regarding left atrial parameters such as LAVI (25.3 ± 8.2% vs. 19.2 ± 7.5%, respectively), AC-CT (34.8 ± 8.6% vs. 44.8 ± 11.8%, respectively), and AC-R (−6.0 ± 4.9% vs. -11.5 ± 3.5%, respectively) were also statistically significant (*p* < 0.01). The assessment of the study group regarding the number of patients with various disturbed diastolic function parameters is summarized in Table S3 in the supplementary file^17,18^.

**Table 3:** Comparison of the diastolic parameters in the study and control groups.

Characteristic	Study group	Control group	MD (95% CI)	*p*
E (cm/s)	89.28 ± 11.65	91.12 ± 14.72	2.16 (−3.87; 8.19)	0.48
Edt (ms)	176.58 ± 54.80	136.94 ± 27.83	39.64 (21.44; 57.84)	** < 0.01**
A (cm/s)	54.59 ± 13.24	53.04 ± 9.13	1.55 (−3.31; 6.41)	0.53
E/A	1.66 (1.54;1.98)	1.69 (1.37;1.96)	−0.03 (−0.15;0.21)	0.84
IVRT (ms)	96.60 ± 19.09	72.86 ± 13.67	23.74 (16.68; 30.81)	** < 0.01**
E' (cm/s)	12.57 ± 2.96	13.37 ± 1.86	−0.80 (−1.84; 0.24)	0.13
A' (cm/s)	6.42 ± 1.68	6.61 ± 1.22	−0.18 (−0.81; 0.44)	0.56
E/E'	7.60 ± 1.81	6.73 ± 1.05	0.87 (0.25; 1.49)	** < 0.01**
LAVI (ml/m^2^)	25.3 ± 8.2	19.2 ± 7.5	6.1 (2.6; 9.6)	** < 0.01**
AC-R (%)	34.8 ± 8.6	44.8 ± 11.8	−10.0 (−14.8; −5.0)	** < 0.01**
AC-CD (%)	−29.0 (−32.0; 23.4)	−32.7 (−39.9; 26.5)	3.6 (−0.01; 9.2)	0.051^1^
AC-CT (%)	−6.0 ± 4.9	−11.5 ± 3.5	5.2 (3.2; 7.2)	** < 0.01**

Significant values are in bold.

*E* early diastolic mitral inflow velocity, *A* atrial diastolic mitral inflow velocity, *Edt* E wave deceleration time, *MD* mean/median differences, *CI* confidential interval, *IVRT* isovolumetric relaxation time, *E*’ early diastolic velocity, *A*’ late diastolic velocity. *MD* mean/median differences, *CI* confidential interval, *LAVI* left atrial volume index, *AC-CT* contractile strain, *AC-R* reservoir strain, *AC-CD* conduit strain.

### Cardiopulmonary exercise test (CPET)

During CPET, patients and controls achieved maximal effort, with RER ≥ 1.05 and a peak heart rate of 85% of the predicted value. In the study group, the resting heart rate was 94 ± 17 bpm, and the maximal heart rate was 186 ± 11 bpm. The maximal oxygen uptake (VO2 max) in the study group reached 33.1 ± 6.2 ml/min/kg in controls. The exact data on CPET parameters in the study and control groups are summarized in Table 4.

**Table 4:** CPET parameters in the study and the control group.

CPET	Study group	Control group	MD/OR (95% CI)	*p*
Rest HR (bpm)	94 ± 17	89 ± 12	6.00 (−0.74; 12.74)	0.10
Maximal HR (bpm)	186 ± 11	188 ± 8	1.4 (−3.4; 5.4)	0.28
SBP (mmHg)	160 ± 27	150 ± 22	5.8 (−6.9; 16.9)	0.15
DBP (mmHg)	70 ± 9	66 ± 11	4.3 (−0.8; 8.8)	0.06
VO2max (ml/min/kg)	33.1 ± 6.2	40.7 ± 6.9	−7.3 (−10.4; −4.2)	** < 0.01**
RER max	1.10 ± 0.05	1.11 ± 0.05	−0.02 (−0.04; 0.05)	0.11

Significant value is in bold.

*OR* odds ratios, *MD* mean/median differences, *HR* heart rate, *SBP* systolic blood pressure, *DBP* diastolic blood pressure, *VO2* oxygen uptake, *O2P* oxygen pulse, *RER* the respiratory exchange ratio.

### Analysis of diastolic echocardiographic parameters regarding the arrhythmia burden and exercise capacity

**Figure 1:**
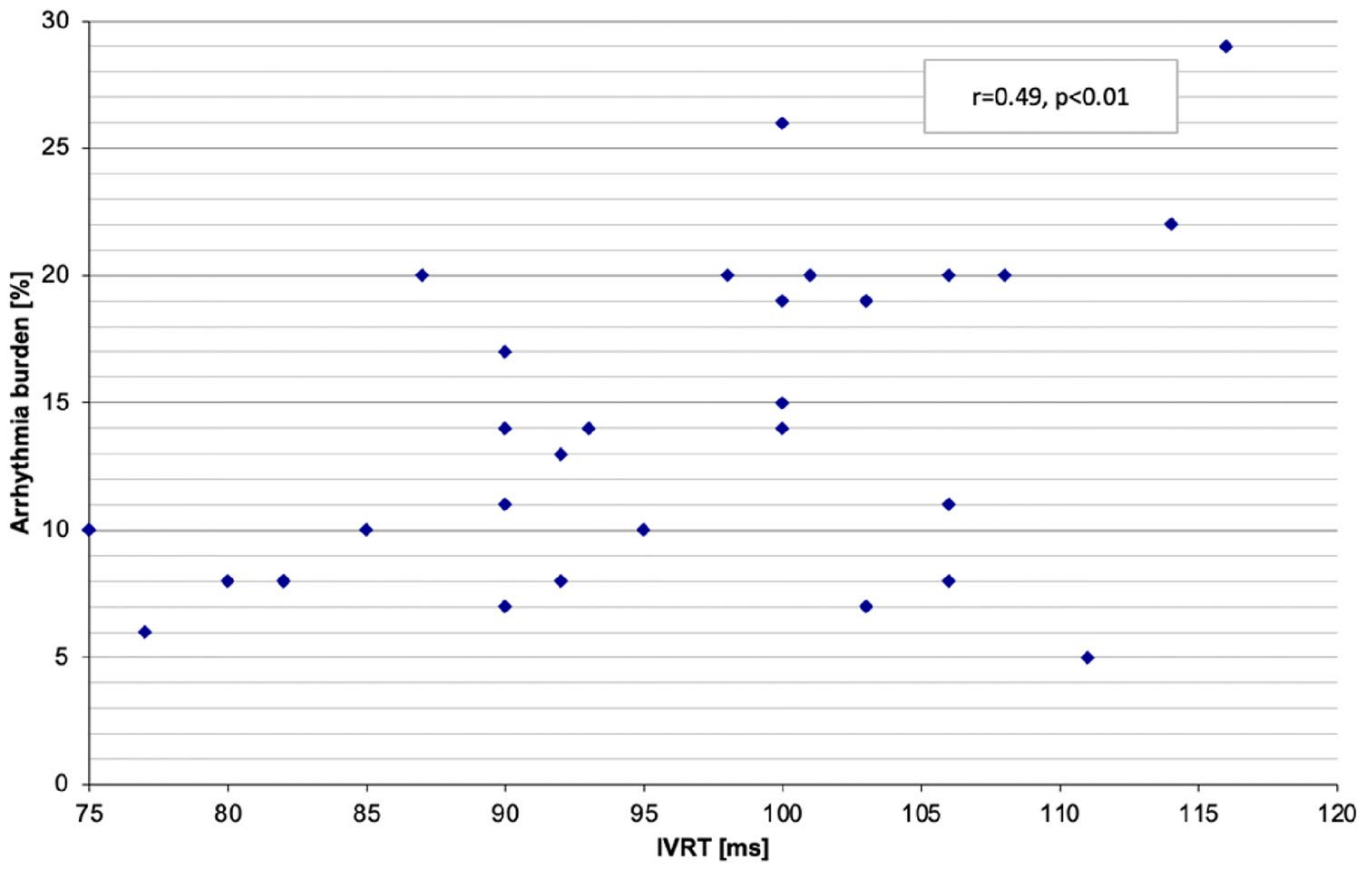
Correlation between the IVRT and the arrhythmia burden. Arrhythmia burden—the percentage of ventricular extra beats in comparison to the number of all beats per 24 h, IVRT—isovolumetric relaxation time.

A moderate, statistically significant, positive correlation was detected between the IVRT and arrhythmia burden (r = 0.49, *p* < 0.01). Figure 1.

**Figure 2:**
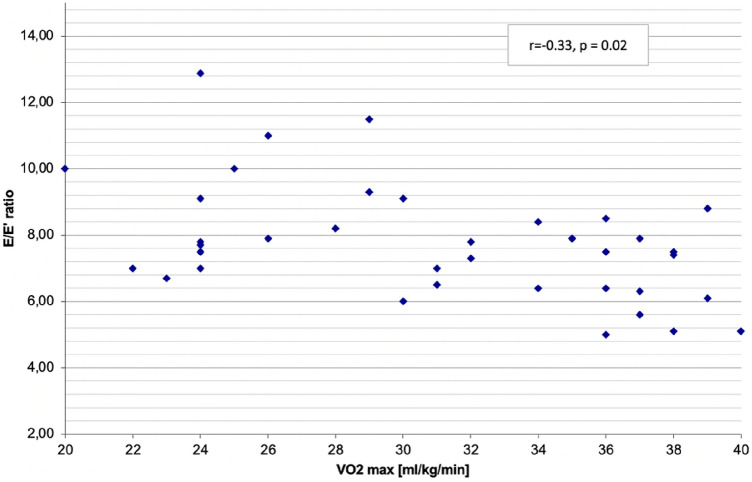
Correlation between the maximal oxygen consumption (VO2max) and E/E’ ratio. E/E’—the ratio of the E wave of mitral inflow measured in the pulse wave doppler to the E’ velocity in the medial site of the mitral annulus measured in the tissue doppler imaging, VO_2max_—maximal oxygen uptake.

Furthermore, a moderate, negative correlation between maximal oxygen consumption (VO2 max) and E/E’ ratio was detected (r = −0.33, *p* = 0.02). Figure 2.

The other correlations between the diastolic function parameters and the arrhythmia burden and maximal oxygen consumption in the study group were both weak and insignificant. Tables S4, S5 supplementary data.

## Discussion

Frequent premature ventricular contractions (PVC) are usually benign. Rarely, they may lead to the contractile dysfunction called “arrhythmic cardiomyopathy”^1^. The risk factors for developing such a condition are defined and listed in the introduction chapter, but the early detection of myocardial function impairment is still challenging. Furthermore, in children, ventricular arrhythmia is usually a primary disease, and the diagnosis of the left ventricular dysfunction before irreversible consequences appear enables proper therapeutic intervention and may substantially improve the prognosis. In our study, we found that diastolic function parameters were disturbed, despite the normal left ventricular systolic function, which suggests that diastolic function disturbances precede systolic myocardial impairment in PVC patients.

Among these parameters, the most obvious in children with ventricular arrhythmia was left atrial enlargement and its functional disturbances expressed as strain parameters (reservoir and contractile strain). The left atrial enlargement was already described in adults with PVC in the study of Park et al*.*^8^, where the indexed left atrial volume correlated statistically significantly with the arrhythmia burden. In our pediatric population, contrary to the study by Park et al*.*, the correlation between arrhythmia burden and left atrial parameters was weak and not statistically significant. There are several explanations for this discrepancy. Firstly, in the cited data, the degree of linear correlation between PVC burden and left atrial size could have been stronger despite a larger study population (146 patients) than ours (36 patients). Secondly, the cited trial was performed in a relatively old population (mean age 55 years) compared to ours (mean age 14 years), so left atrial size, reflecting left ventricular filling pressure, may relate not only to PVC burden but also to the low compliance of the myocardium due to its degeneration, which could to some extent empower increased filling pressure associated with the ventricular arrhythmia.

Furthermore, we found statistically significant differences between the controls and patients regarding left atrial strain variables. The cut-off limits of left atrial strains for children are not established, so the meaning of our findings is uncertain. Further detailed evaluation on a bigger cohort would detect cut-off limits of left atrial strains for PVC children and shed light on the clinical value of these findings.

The elongation of the E deceleration time (Edt) in our study group suggests that ventricular diastolic dysfunction is at the disease’s initial stage. In general, Edt preservation is closely connected with diastolic function impairment severity. In adults, the elongation of Edt is often seen as a physiological process due to the gradual decline of the myocardial compliance with age, but in young humans it reflects early diastolic left ventricular stiffness due to mildly impaired relaxation. Pseudo-normal or mildly reduced Edt is a sign of concomitant concentric left ventricular hypertrophy, and markedly reduced Edt means restrictive filling and advanced diastolic dysfunction^19,20^.

In our patients, the elongation of Edt was probably observed as a reflection of mild relaxation disturbances. We can only speculate that in some patients, during long-term follow-up, the diastolic function would deteriorate further, leading to the irreversible stiffness of the ventricle if proper antiarrhythmic treatment was not performed.

To some point, isovolumetric relaxation time (IVRT) analysis confirms our hypothesis. IVRT was also found to be elongated in our population, which, to the best of our knowledge, was never described in the literature concerning PVC in children. Its increase relates to early diastolic dysfunction in many diseases such as hypertrophic cardiomyopathy, arterial hypertension, or diabetes^21,22^ In PVC children, it is the consequence of dyssynchronous contraction due to arrhythmia, which leads in a natural way to dyssynchronous relaxation. The dyssynchronous myocardial activation is seen not only when a premature beat occurs but also during sinus rhythm neighboring the premature contraction^23^. It probably normalizes gradually from beat neighboring to beat remote from PVC. The evidence for the last assumption does not exist in the literature and needs some further investigation. Still, it could explain why IVRT relates to the arrhythmia burden in our patients.

Another diastolic function parameter disturbed in adolescents with PVCs is the E/E’ ratio. It was significantly higher in the cases than in the controls. E/E’ is the primary factor reflecting the filling pressure of the left ventricle, which, according to the guidelines, has become one of the essential noninvasive diastolic function parameters^20^. Our results are concordant with the study of Salem et al*.*^7^ in which it was significantly higher in adults with PVC than in the controls. Moreover, our recent trial found a statistically significant correlation between exercise capacity and E/E’. The elevated filling pressure of the left ventricle is strictly related to the low exercise capacity in many diseases, e.g., aortic stenosis, atrial fibrillation, or diabetes^10–13^. Considering such data, we may assume that high E/E’ in children with PVC may reflect increased filling pressure, which probably does not normalize during effort and directly influences exercise capacity. Another possible explanation is that dyssynchronous contraction is the primary factor connected with the low exercise capacity, and the diastolic filling pressure is less meaningful for the exertion tolerance in PVC patients. We can assume that further analysis using detailed hemodynamic evaluation, e.g., stress echocardiography, would help explain the mechanism of low exercise capacity in young individuals with ventricular heart rhythm disturbances.

In conclusion, left ventricular diastolic function is impaired and deteriorates with the arrhythmia burden increase in PVC children. Ventricular arrhythmia in young individuals may be related to exercise capacity decrease due to the left ventricular filling pressure elevation.

## Limitations

In our study, we did not precisely perform the modified Borg RPE score and did not use it as an inclusion criterion. In our opinion, self-assessment of the exertion level is frequently inadequate in the pediatric population. However, our department has a routine clinical practice to evaluate the level of perceived exertion during the CPET. The test is valid only if the modified Borg RPE score is 7/10. All the patients in the study group and the controls had valid tests, which means that all of them achieved at least grade 7 on the modified Borg RPE scale.

We did not evaluate an exact number of hours of activity. Nonetheless, all the patients and controls had a similar level of everyday physical training of up to 4 h of recreational sport per week at school. We also did not record the exact results of laboratory tests such as the blood cell count and ion balance. Basic laboratory tests were performed in all patients due to clinical evaluation, and all the results were normal or insignificant from the clinical point of view.

This is a pilot study. A detailed analysis of the diastolic function in a larger cohort of patients with ventricular arrhythmia is necessary to confirm our findings. Moreover, the significant correlations between chosen parameters were only moderate in our patients. Thus, it needs to be confirmed in the future whether any link between diastolic dysfunction and physical performance and arrhythmia burden in PVC children exists.

## Supplementary Information

Supplementary Information.

## Data Availability

Data can be available under reasonable request. If someone wants to request the data from this study, a corresponding author Radosław Pietrzak should be contacted; e-mail: radoslaw.pietrzak@wum.edu.pl.
