# Supplementary material for: Diastolic function evaluation in children with ventricular arrhythmia
Source: Sci Rep. 2023 Apr 11;13:5897. doi: 10.1038/s41598-023-33118-x (PMC10090147; doi:10.1038/s41598-023-33118-x)
Supplement: Supplementary file 1 — Supplementary Information. [file 41598_2023_33118_MOESM1_ESM.docx]

*Table S1. Reliability of correlations scale used in the trial according to the Stanisz scale.*

| \|r\|=0: lack of correlation 0,0<\|𝑟\|≤0,1 very weak  0,1<\|𝑟\|≤0,3 weak  0,3<\|𝑟\|≤0,5 moderate 0,5<\|𝑟\|≤0,7 strong 0,7<\|𝑟\|≤0,9 very strong 0,9<\|𝑟\|<1,0 almost perfect \|𝑟\|=1 perfect. |
| --- |

*Table S2. ECG parameters in the control and the study group.*

| ECG parameter | Study group | Control group | p |
| --- | --- | --- | --- |
| HR (bpm) | 75±12 | 74±12 | 0.59 |
| PR (ms) | 130 ±15 | 137 ±19 | 0.24 |
| QRS (ms) | 78±13 | 77±8 | 0.21 |
| QT (ms) | 363±19 | 62.2±6.4 | 0.14 |
| QTc (ms) | 399±20 | 395±15 | 0.55 |
| 24- hour Holter monitoring parameters |  |  |  |
| Mean value of bpm | 88±12 | 89±19 | 0.83 |
| Minimal value of bpm | 55±11 | 57±13 | 0.85 |
| Maximal value of bpm | 143±27 | 147±22 | 0.63 |

*bpm - beats per minute, HR- heart rate, PR- PR interval, QRS – QRS complex, QT – QT interval, QTc – QT interval corrected according to the Bazett formula.*

Table S3. Presentation of the study population regarding diastolic dysfunction.

| Disturbed parameter | Number of patients |
| --- | --- |
| IVRT | 3 |
| E/E’ | 3 |
| LAVI | 3 |
| Edt | 5 |
| IVRT + E/E’ | 2 |
| IVRT + LAVI | 2 |
| IVRT + Edt | 1 |
| E/E’ + Edt | 1 |
| Edt + LAVI | 1 |
| IVRT + Edt + E/E’ | 4 |

*E – E wave velocity, Edt – E wave deceleration time, IVRT – isovolumetric relaxation time, E’ - early diastolic velocity, LAVI – left atrial volume indexed to the body surface area.*

*Table S4. Correlation analyses between those parameters of the diastolic function which significantly differentiated the study group and controls, and the arrhythmia burden*

| Characteristic | Arrhythmia burden | |
| --- | --- | --- |
|  | r | p |
| Edt | 0.24 | 0.09 |
| E/E' | 0.19 | 0.18 |
| LAVI | 0.15 | 0.29 |
| AC-CT | -0.1 | 0.55 |
| AC-R | 0.1 | 0.61 |

*Edt – E wave deceleration time, IVRT – isovolumetric relaxation time, E’ - early diastolic velocity, LAVI – left atrial volume indexed to the body surface area, AC-CT contractile strain AC-R reservoir strain.*

*Table S5. Correlation analyses between those parameters of the diastolic function which significantly differentiated the study group and controls and the maximal oxygen consumption in the study group (VO2max)*

| Characteristic | VO2 max | |
| --- | --- | --- |
|  | r | P |
| Edt | 0.22 | 0.14 |
| IVRT | -0.06 | 0.66 |
| LAVI | -0.16 | 0.27 |
| ACT-CT | -0.18 | 0.20 |
| ACT-R | 0.11 | 0.85 |

*Edt – E wave deceleration time, IVRT – isovolumetric relaxation time, E’ - early diastolic velocity, LAVI – left atrial volume indexed to the body surface area, AC-CT contractile strain AC-R reservoir strain.*
